# Impact of zero-markup consumable policy and national procurement of orthopedic spinal devices in China: a mixed-methods study

**DOI:** 10.3389/fpubh.2025.1715856

**Published:** 2026-01-21

**Authors:** Xiaojuan Wu, Xue Yao, Qingxiang Zhao, Xuejie Qi, Yiyu Zhang, Xianzhu Cong, Yue Huang, Xiao Qi, Fuyan Shi, Suzhen Wang

**Affiliations:** 1 Department of Health Statistics, School of Public Health, Shandong Second Medical University, Weifang, China; 2 Zibo 148 Hospital, Zibo, China; 3 Department of Pain Management, Binzhou Medical University Hospital, Binzhou, China

**Keywords:** centralized volume-based procurement, consumables pricing reform, mixed-methods design, regression discontinuity design, zero markup consumable policy

## Abstract

**Background:**

Since 2019, Chinese has launched a nationwide pricing reform targeting medical consumables in the public hospitals, aimed at reducing costs and alleviating the financial burdens. The key initiatives include the Zero Markup Consumables Policy (ZMCP, Reform1), implemented in 2019, and the Centralized Volume-Based Procurement (CVBP, Reform2) for orthopedic spinal devices, initiated in Shandong province in 2023.

**Objectives:**

The study aims to assess the impacts of the ZMCP and CVBP reforms on hospitalization expenditures and clinical outcomes for orthopedic spinal inpatients, and to identify challenges associated with policy implementation.

**Methods:**

Data concerning orthopedic spinal inpatients was extracted from Binzhou Medical University Hospital in China, the retrospective period spanning from January 2018 to June 2024. A mixed-methods design approach was adopted, combining a regression discontinuity design (RDD) (*n* = 1,099) with semi-structured interviews (*n* = 7) to comprehensively assess the impacts of the reforms.

**Results:**

The study provides evidence that the expenses for total hospitalization and medical consumables demonstrated a significant decrease after the CVBP reform, dropping from 18,288.08 CNY (pre-reform) to 11,461.09 CNY (Reform2) and from 8,718.27 CNY (pre-reform) to 1,178.83 CNY (Reform2), respectively. Expenses in other categories did not show statistically significant changes during the reforms. The proportion of expenses for medical consumables decreased significantly, from 47.07% (pre-reform) to 43.27% (Reform1), and further to 10.19% (Reform2). The postoperative length of stay (post-LOS) and postoperative numerical rating scale (post-NRS) showed no statistically significant changes during the reform periods. The qualitative findings revealed that most notably financial strain on hospitals and policy-driven shifts in clinical decision-making.

**Conclusion:**

The study suggests that following the implementation of the CVBP policy in Chinese public hospitals, pricing reforms for medical consumables were associated with reductions in both medical consumable and total hospitalization expenditures, as well as a shift in the composition of expenses. In contrast, the reform of ZMCP appeared to have limited measurable impact within the specific context of this study. By integrating quantitative and qualitative evidence, this research helps to clarify the specific complexities and challenges involved in implementing health policy reforms in China, and it provides preliminary insights into the potential mechanisms driving these observed changes.

## Graphical abstract

**Figure:**
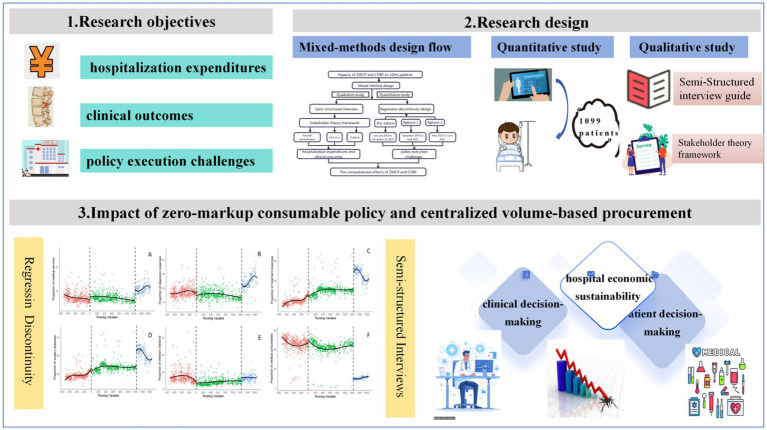

## 1 Background

The escalating trend in hospitalization costs presents significant challenges to healthcare systems globally (1). In China, the growth of total health expenditures has been particularly notable, expanding at an average annual rate of 12.2% between 2009 and 2019—this rate notably exceeds the country’s GDP growth, which stood at 8.1% during the same period (2). The complexity of rising medical expenditures in China is further exacerbated by the unique public hospital financing mechanisms, deeply rooted in the historical context of the nation’s health systems (3–5).

Following market-oriented reforms initiated in the 1980s (4, 6, 7), subsidies to public hospitals markedly decreased from 40 to 10% (8). This reducing led to the implementation of markup policies on drugs and consumables (15% for drugs, 10% for consumables) as alternative sources of revenue (9). While these policies were intended to ensure the financial viability of hospitals, they inadvertently contributed to the increase in medical expenditures.

In response to these persistent challenges, China embarked on comprehensive reforms of its healthcare system targeted at public hospitals in 2009 (10). These reforms included two significant initiatives regarding medical consumables: ① The elimination of markups on drugs and medical consumables coupled with adjustments in the pricing of medical services; ② The implementation of national centralized procurement programs focusing on high-value medical consumables (11). These policies are being progressively implemented across all provinces and cities within China. Shandong Province experienced two distinct phases of comprehensive reforms concerning the pricing of consumables in public hospitals in 2019 and 2023, respectively. The initial phase in 2019 emphasized the implementation of a zero markup consumables policy (ZMCP). Subsequently, in its second phase, the Chinese government progressively promoted the centralized volume-based procurement (CVBP) of high-value medical consumables, including coronary stents and artificial joints. Motivated by the escalating demand for addressing lumbar disc herniation (LDH) in China (12, 13), the third wave of centralized procurement incorporated orthopedic spinal devices for percutaneous endoscopic lumbar discectomy (PELD) procedures (14).

Despite these significant policy initiatives, critical evidence gaps remain in China. First, existing scholarship has predominantly concentrated on pharmaceutical policies (15, 16), with significantly less attention paid to medical consumables, particularly those used in orthopedic spinal applications. Moreover, the centralized procurement of medical consumables has imposed limitations on the evaluation of clinical efficacy. Third, previous studies have largely depended on either quantitative assessments (17) or policy-oriented analyses (18), thus offering limited holistic appraisals of the policy’s repercussions.

Guided by this context, the present study employed a sequential explanatory mixed-methods design to investigate the following research questions:

1. What was the quantitative impact of the medical consumables reform on patient expenditure, and clinical outcomes among orthopedic spinal inpatients?
2. How did the policy, as perceived by key stakeholders, affect the operational dynamics and clinical decision-making processes in hospitals?
3. How do the qualitative findings elucidate and corroborate the quantitative results, thereby facilitating a more comprehensive understanding of the policy’s multifaceted effects?

Observational data of quantitative analysis collected from Binzhou Medical University Hospital (Shandong, China) from January 2018 to June 2024 (*N* = 1,099) were utilized to examine alterations in medical expenditures and the challenges faced by hospitals in implementing the ZMCP and CVBP. Qualitative analysis was conducted based on semi-structured interviews with seven key informants, including public hospital administrators, clinicians, and patients.

## 2 Methods

### 2.1 Mixed-methods design

**Figure 1:**
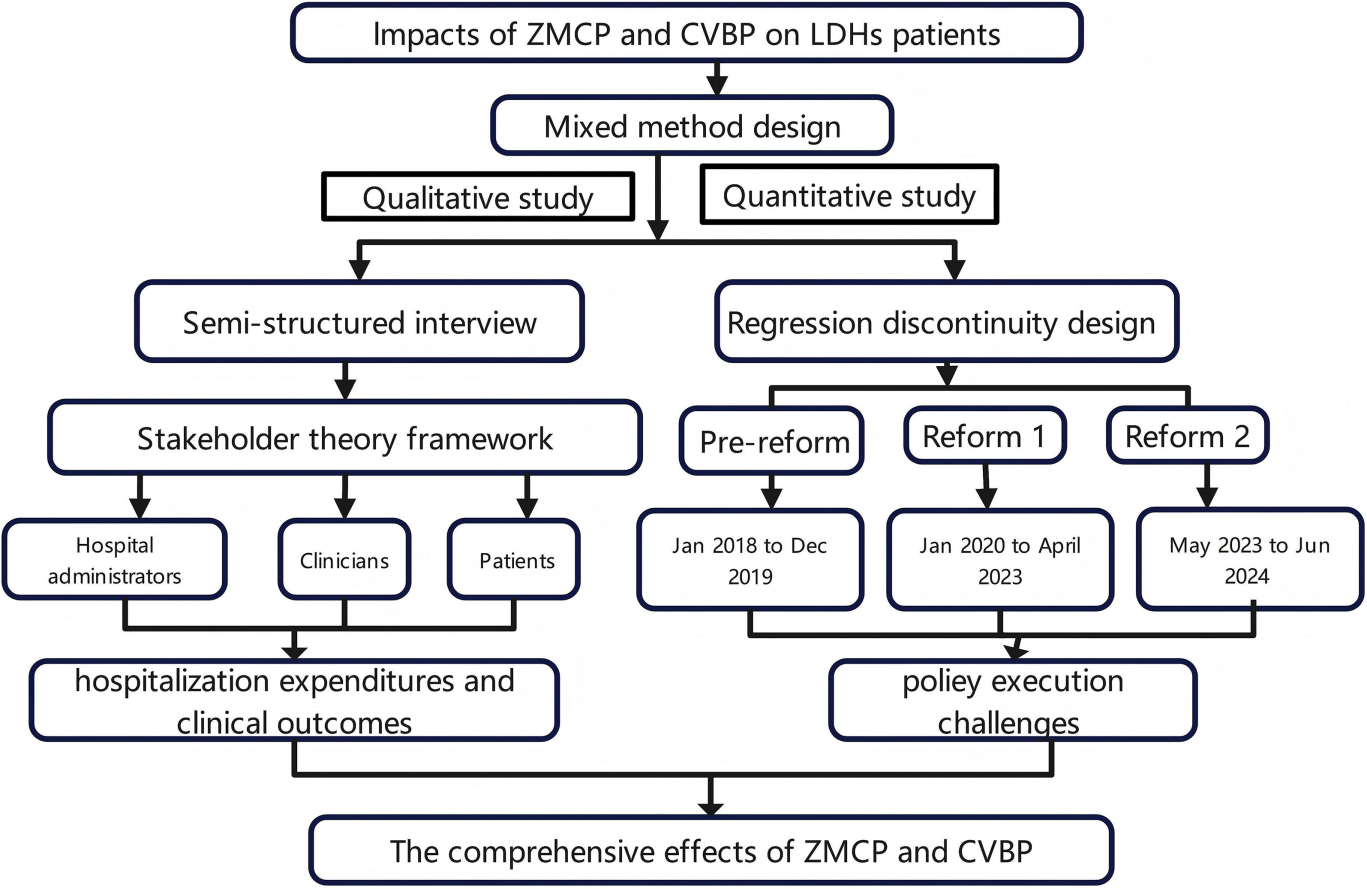
Mixed methods design research flow chart.

A sequential explanatory mixed-methods design was employed to evaluate the impact of the consumables price policy in China, as illustrated in Figure 1. Mixed methods (19), quantitative study combining with qualitative one, were selected to assess the comprehensive effects of ZMCP and CVBP. The qualitative phase was explicitly designed to build upon the initial quantitative results. For the quantitative study, RDD was used to evaluate the medical expenses for patients undergoing PELD before and after the implementation of the reforms. Key outcomes from the RDD were presented to qualitative interviewees. For the qualitative component, in-depth interviews were conducted with key informants selected through purposive sampling to provide additional supporting evidence explaining the underlying reasons and mechanisms behind the observed cost changes, thereby enriching the quantitative findings and contextualizing.

### 2.2 Quantitative study

#### 2.2.1 Data

This retrospective cohort study assessed 1,099 patients diagnosed with LDH at Binzhou Medical University Hospital (Shandong, China) from January 2018 to June 2024. The inclusion criteria were as follows: ① Patients diagnosed with LDH as confirmed by MRI (ICD -10: M51.2 - M51.9), ② All patients who underwent a primary PELD procedure (ICD - 9 - CM - 3: 80.51), and ③ Patients with complete clinical documentation. The exclusion criteria included: ① Patients who underwent other spinal procedures or revision surgeries; ② Patients who required additional surgery during hospitalization for other conditions; ③ Patients who were unable to tolerate surgery due to severe comorbidities; and ④ Patients with incomplete data. The participant flow chart is presented in Supplementary Figure S1. Comprehensive data on medical expenditures, demographic characteristics, and clinical outcomes were extracted for each participant from electronic medical records. The study cohorts were stratified into three groups according to the timelines of policy implementation: the pre-reform group (*n* = 399), spanning from January 2018 to December 2019; the Reform phase1 group (*n* = 534), covering the period from January 2020 to April 2023 during the implementation of the ZMCP; the Reform phase2 group (*n* = 166), from May 2023 to June 2024 following the introduction of the CVBP.

#### 2.2.2 Variables

The study focused on two primary outcomes: inpatient hospitalization expenditure and clinical efficacy. Hospitalization expenditure was categorized into six distinct groups: ① Expenses for comprehensive medical services; ② Expenses for Western medicine; ③ Diagnostic expenses which include pathology diagnostic expenses, laboratory diagnostic expenses, imaging diagnostic expenses, and clinical diagnostic project expenses; ④ Treatment expenses, encompassing general treatment operation expenses, non-surgical treatment project expenses, and general medical service expenses; ⑤ Surgical expenses; ⑥ Expenses for medical consumables which cover disposable medical consumables used in examinations and treatments. The proportions of expenditure were calculated though dividing the total expenses in each category by the total hospitalization expenses. Clinical efficacy was assessed using two variables: ① The postoperative numerical rating scale (post-NRS), utilized to measure the degree of pain relief following surgery (20); ② The postoperative length of stay (post-LOS), which serves as an indicator of recovery (21). The dataset used in the analysis was complete, with no missing values for any variables.

#### 2.2.3 Regression discontinuity design

In the quantitative analysis, the RDD was utilized to estimate the causal effects of the intervention before and after the reform (22). The RDD was employed when individuals were allocated to an exposure based on whether they fall above or below a predetermined threshold on a continuous scale.

**Figure 2:**
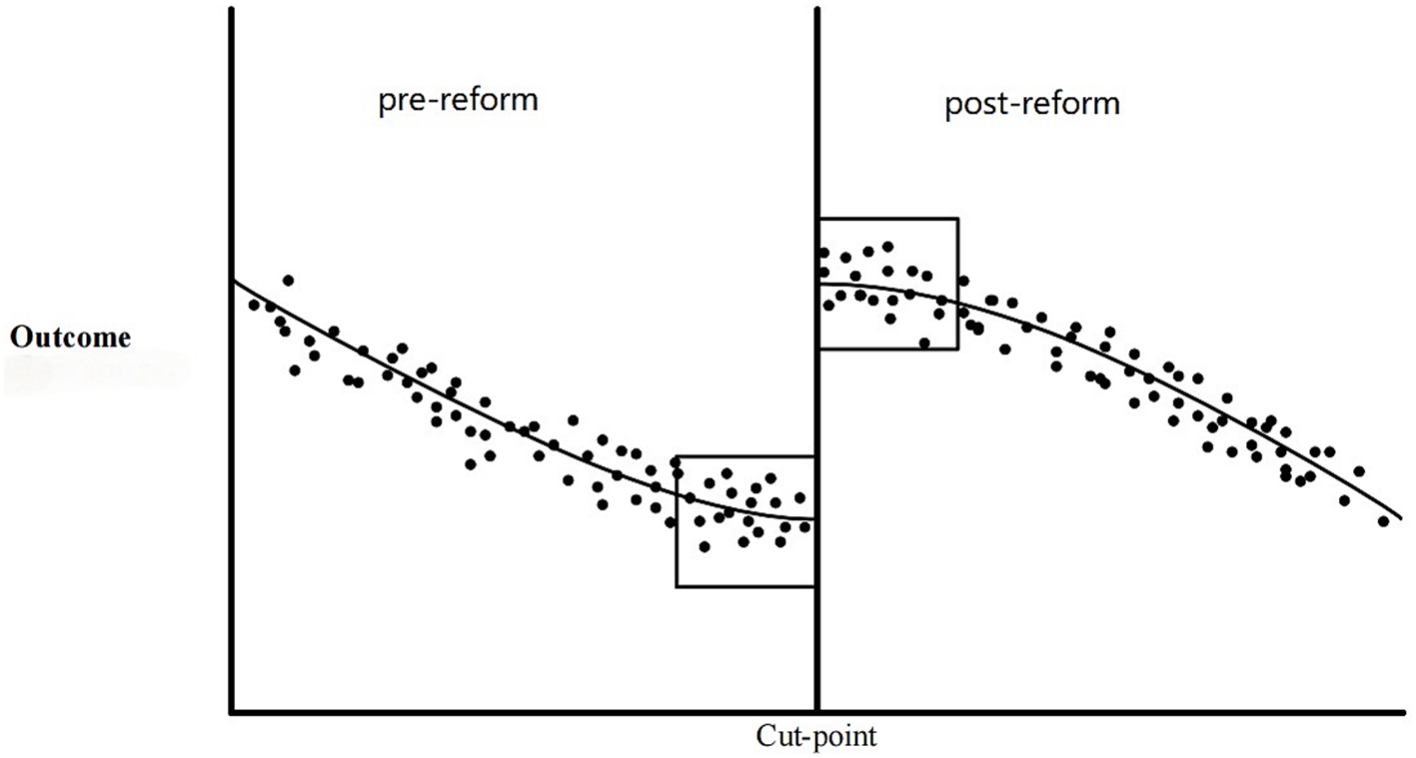
Graphical representation of the regression discontinuity design. Dots represent individual patients. The vertical line in the center of each graph designates a cut-point, above which candidates are assigned to the treatment and below which they are not. The boxes represent the proportion of the distribution proximal enough to the cut-point to be used in regression discontinuity analysis, assuming local randomization (51).

Consequently, the probability of exposure changes abruptly at the threshold, which depends on the continuous underlying variable. This variable is often referred to as the running variable in RDD studies. The RDD analysis evaluates any sudden shifts in the likelihood of the outcome at the same threshold. The magnitude of this discontinuity is used to estimate the causal effects of the policy change or intervention on those nearby. Figure 2 illustrates the application of the RDD in this study (23). The vertical cut-point strictly determines treatment assignment. Patients discharged after this date are categorized into the post-reform group, whereas those discharged prior to this date constitute the pre-reform control group. The cut-point determines treatment assignment; patients discharged after the cut-point are post-reform, while those discharged before it are pre-reform. The observed jump in the outcome at the threshold indicates the local causal effect of the reform, assuming continuous variation of other factors.

The running variable is defined as time (in days) relative to the exact implementation date of the policy. For ZMCP, this date is December 30, 2019; for CVBP, it is April 30, 2023. The thresholds for running variable (time relative to the December 30 2019, reform1) were operationally defined as follows: 0 days for ZMCP; and 1,236 days for CVBP, with a temporal support ranging from −714 to +1,655 days relative to policy implementation. This establishes a deterministic treatment assignment rule based solely on whether the running variable crosses the threshold, fulfilling the requirements of a sharp RDD (51). A critical concern in time-based RDD was that the observed discontinuity might be confounded by unrelated temporal shocks. The selected cutoff points were the official, nationwide enforcement dates mandated by the Chinese government for these specific reforms. This design isolates estimated policy effects from unrelated trends using exogenous cutoffs.

The RDD incorporates local polynomial regression with triangular kernel weighting, Mean Square Error (MSE)-optimal bandwidth selection, and robust bias-corrected inference to mitigate boundary effects (24). We adopted the MSE-optimal bandwidth selection procedure formed by Calonico (22). The “MSE-optimal” bandwidth is chosen to minimize the MSE of the local polynomial point estimator. To be specific, it algorithmically selects a bandwidth that reduces the sum of the squared bias and the variance of the estimate. RDD capitalizes on “thresholds” that naturally arise from continuous variables, referred to as “running variables” to assign treatment or intervention RDD (24, 25). The study analyzed ZMCP and CVBP reforms with defined implementation dates for RDD analysis (26). Consequently, the study utilizes a multiple RDD (27, 28) to evaluate the impact of these two policy reforms. Logarithmic transformations were applied to variables that failed normality and homoscedasticity assumptions, such as total hospitalization. Additionally, several indicators were used as control variables to perform covariate-adjusted sharp RDD (24, 25). These indicators comprised LOS, sex and age. The RDD model is specified as follows in Equation.


$$\begin{array}{c} Y_{i}=\beta_{0}+\beta_{1}(X_{i}-c_{1})*D[c_{1}\leq X_{i}\leq c_{2}]+\beta_{2}(X_{i}-c_{1})\\ {}*D[c_{2}\leq X_{i}\leq c_{3}]+\gamma_{1}\mathrm{se}x_{i}+\gamma_{2}\mathrm{LO}S_{i}+\gamma_{3}\mathrm{ag}e_{i}+\varepsilon_{i} \end{array}$$

In this model, the dependent variable $Y_{i}\;$ represents the outcome variable, such as hospitalization expenditure and the proportion of expenses; $X_{i}$ denotes the running variable; $c_{i}$ is the cutoff points; the indicator function D[ $X_{i}$ ] assigns 1 if the condition is true and 0 if false; LOS, sex and age were included as covariates; $\beta_{0}$ is the intercept, $\beta_{1}$ , $\beta_{2}$ ... $\beta_{k}$ represent the intervention effect at each cutoff point, and $\varepsilon_{i}$ is the error term.

Statistical analyses were conducted using R software (version 4.4.1) with the following specialized packages including “rdrobust” “ggplot2” “dplyr” “lubridate” “gridExtra” “segmented” and “rddensity”.

#### 2.2.4 The robustness tests

To validate the robustness of the RDD results, we performed several diagnostic tests following established methodologies (24, 29). Initially, the McCrary density test (30) was employed to confirm a smooth distribution of the running variable and covariates near the cutoff, thereby ruling out significant discontinuities that could undermine the identification assumptions. The test confirmed that the density of the running variable and covariates was statistically continuous at the policy cutoff, supporting the validity of the local randomization assumption. Subsequently, we conducted bandwidth sensitivity analyses (31) to evaluate the stability of estimates across varying bandwidths, thus supporting the robustness of the effects observed. Lastly, we implemented placebo tests (32) using artificial cutoff points to validate our primary results. These tests rigorously address the assumptions underpinning RDD and strengthen the validity of causal inference.

### 2.3 Qualitative study

To comprehensively evaluate the impact of the policy on both patients and public hospitals, we conducted semi-structured interviews with key stakeholders from October to November 2024.

#### 2.3.1 Participant recruitment

Using a heterogeneous purposive sampling approach (33), we aimed to capture diverse perspectives from three key stakeholder groups (34): (1) hospital administrators (e.g., directors of medical equipment or finance departments), (2) clinicians (orthopedic surgeons with experience in spinal surgeries), and (3) patients who had undergone spinal surgery during the study period. Participants were recruited through multiple channels: the hospital administration team assisted in identifying and contacting administrators and clinicians, and patients were referred by their attending clinicians. Guided by the stakeholder theory (35), we developed a semi-structured interview guide aimed at assessing the impact of the consumables policy on these key groups. All interviews were conducted by the same researcher in private settings, lasted between 30 and 40 min, and were audio-recorded with participants’ consent. All recordings were professionally transcribed verbatim in Chinese, and the transcripts were systematically checked against the original recordings to ensure accuracy. The interview guide (Supplementary Table S1) was initially prepared in Chinese and subsequently was translated into English.

#### 2.3.2 Data analysis

Qualitative data analysis was conducted using NVivo 14, where codes were systematically derived from the interview transcripts through an iterative thematic analysis process. To maintain the validity and consistency of our thematic analysis, we employed a strict team-based strategy. The researchers created a codebook, coded transcripts independently, and held meetings to ensure coding reliability.

## 3 Results

### 3.1 Quantitative analysis

#### 3.1.1 Demographic information

This study encompassed a total of 1,099 patients, distributed among three groups: 399 in the pre-reform group, 534 in the Reform phase1 group, and 166 in the Reform phase2 group. The cohort comprised 552 males (50.23%) and 547 females (49.77%). The age distribution indicated that participants aged 45–54 years constituted the largest demographic segment (27.93%), followed by those aged 55–64 years (27.39%) and 65–74 years (22.93%). Conversely, the youngest cohort (15–24 years) represented a mere 1.46% of the sample. The preoperative numeric rating scale (pre-NRS) indicated that 4.46% of patients experienced mild pain, 88.81% moderate pain, and 6.73% severe pain, thereby demonstrating a predominance of moderate pain across all groups. The median LOS decreased progressively across the reform periods, from 11.00 days in the pre-reform phase to 10.00 days in Reform1 and further to 9.00 days in Reform2 (*p* < 0.001; Table 1). There were no significant differences in demographic variables such as age, sex, or pre-NRS scores among the three groups.

**Table 1:** Demographic information of LDH patients.

Variable	Before-reform (*N* = 399)	Reform phase 1 (*N* = 534)	Reform phase 2 (*N* = 166)	Total	*p*-value
Sex (*N*, %)					
Male	195 (48.87)	265 (49.63)	92 (55.42)	552 (50.23)	0.339
Female	204 (51.13)	269 (50.37)	74 (44.58)	547 (49.77)	
Age (*N*, %)					
15–24 years	7 (1.75)	7 (1.31)	2 (1.20)	16 (1.46)	0.261
25–34 years	20 (5.01)	21 (3.93)	6 (3.61)	47 (4.28)	
35–44 years	43 (10.78)	53 (9.93)	22 (13.25)	118 (10.74)	
45–54 years	108 (27.07)	163 (30.52)	36 (21.69)	307 (27.93)	
55–64 years	111 (27.82)	142 (26.59)	48 (28.92)	301 (27.39)	
65–74 years	81 (20.30)	129 (24.16)	42 (25.30)	252 (22.93)	
75- years	29 (7.27)	19 (3.56)	10 (6.02)	58 (5.28)	
Pre-NRS					
Mild pain	21 (5.26)	22 (4.12)	6 (3.61)	49 (4.46)	0.850
Moderate pain	352 (88.22)	477 (89.33)	147 (88.55)	976 (88.81)	
Severe pain	26 (6.52)	35 (6.55)	13 (7.83)	74 (6.73)	
LOS, M (IQR)	11.00 (4.00)	10.00 (3.00)	9.00 (2.00)	11.00 (4.0)	<0.001*

Pre-NRS, Preoperative Numeric Rating Scale; LOS, Length of Stay; M, Median; IQR, Inter-Quartile Range. Mild pain: 0–3; Moderate pain: 4–6; Severe pain: 7–10. Between-group comparisons were made using: Sex, Age, and Pre-NRS: Chi-squared test; LOS: Kruskal-Wallis rank test. *p < 0.05.

This finding demonstrates that the groups are equivalent at baseline, which is essential for accurately observing differences in policy effects. This baseline equivalence strengthens the credibility of causal inferences drawn from the quasi-experimental design.

#### 3.1.2 Descriptive analysis

The total hospitalization expenses for patients showed a marginal increase from 18,288 (IQR, 2439) CNY to 18,312 (IQR, 2,975) CNY following the implementation of the ZMCP policy. In contrast, expenses decreased significantly by 6,827 CNY, from 18,288 (IQR, 2,439) CNY to 11,461 (IQR, 2,111) CNY, subsequent to the CVBP policy. Specifically, treatment expenses escalated from 3,482 (IQR, 728) CNY to 5,649 (IQR, 965) CNY in Reform1 and to 5,734 (IQR, 2,022) CNY in Reform2. Similarly, surgery expenses rose from 3,330 (IQR, 607) CNY to 4,990 (IQR, 918) CNY in Reform1 and to 5,480 (IQR, 2000) CNY in Reform2. In contrast, expenditures on Western medicine dropped from 1,130 (IQR, 643) CNY to 704 (IQR, 456) CNY in Reform1 and to 726 (IQR, 284) CNY in Reform2; expenditures on medical consumables also decreased from 8,718 (IQR, 295) CNY to 7,669 (IQR, 199) CNY in Reform1 and further to 1,178 (IQR, 99) CNY in Reform2.

The proportion of medical consumables decreased consistently, while the proportions of other expense categories increased. Specifically, the proportion of medical consumables decreased from 47.07% (IQR, 6.29%) to 43.27% (IQR, 5.2 6%) in Reform1 and to 10.19% (IQR, 1.74%) in Reform2. Concurrently, the proportion of treatment expenses rose from 18.84% (IQR, 4.08%) to 29.62% (IQR, 4.66%) in Reform1 and to 47.81% (IQR, 10.02%) in Reform2, and the proportion of surgery expenses increased from 17.96% (IQR, 3.53%) to 26.99% (IQR, 3.94%) in Reform1 and to 43.38% (IQR, 11.01%) in Reform2. Clinical outcome variables, including post-LOS and post-NRS scores, remained relatively stable (Table 2). The median post-LOS was reduced from 8 days to 7 days in Reform1 and remained at 7 days in Reform2. The post-NRS was consistently 0 during the reform periods. The analysis reveals that the ZMCP policy had little effect on hospitalization costs, while the CVBP policy significantly reduced overall spending; both policies decreased consumable costs but increased treatment costs, with no impact on clinical outcomes like post-LOS and post-NRS.

**Table 2:** The statistical description expenditures and clinical outcomes.

Outcome		Jan 2018 to Dec 2019 (before-reform)	Jan 2020 to Apr 2023 (Reform phase 2)	May 2023 to Jun 2024 (Reform phase 2)
Expenses, M(IQR), CNY	Total hospitalization expenses	18288.08 (2439.90)	18312.38 (2975.70)	11461.09 (2111.33)
	Medical service expenses	1208.00 (456.50)	1234.50 (408.00)	1150.50 (285.50)
	Diagnostic expenses	3060.00 (866.00)	2729.45 (784.18)	2653.60 (588.75)
	Treatment expenses	3482.50 (727.75)	5649.00 (965.00)	5734.00 (2022.50)
	Surgery expenses	3330.00 (607.00)	4990.50 (918.00)	5480.00 (2000.00)
	Western medicine expenses	1130.92 (643.07)	704.55 (456.24)	726.92 (284.71)
	Medical consumable expenses	8718.27 (295.25)	7669.40 (199.81)	1178.83 (99.98)
Proportion of expenses, M (IQR), %	Proportion of medical services expenses	6.66 (2.04)	6.79 (1.85)	10.59 (2.77)
	Proportion of diagnostic expenses	16.61 (3.91)	14.92 (3.85)	23.57 (6.69)
	Proportion of treatment expenses	18.84 (4.08)	29.62 (4.66)	47.81 (10.02)
	Proportion of surgery expenses	17.96 (3.53)	26.99 (3.94)	43.38 (11.01)
	Proportion of Western medicine expenses	6.32 (3.27)	3.84 (1.95)	6.395 (2.14)
	Proportion of medical consumables	47.07 (6.29)	43.27 (5.26)	10.19 (1.74)
Clinical outcomes, M(IQR)	Post-NRS	0 (1)	0 (1)	0 (1)
	Post-LOS	8 (2)	7 (1)	7 (0.75)

Post-NRS, Postoperative numerical rating scale; Post-LOS, Postoperative length of stay.

#### 3.1.3 RDD validation and main results

**Figure 3:**
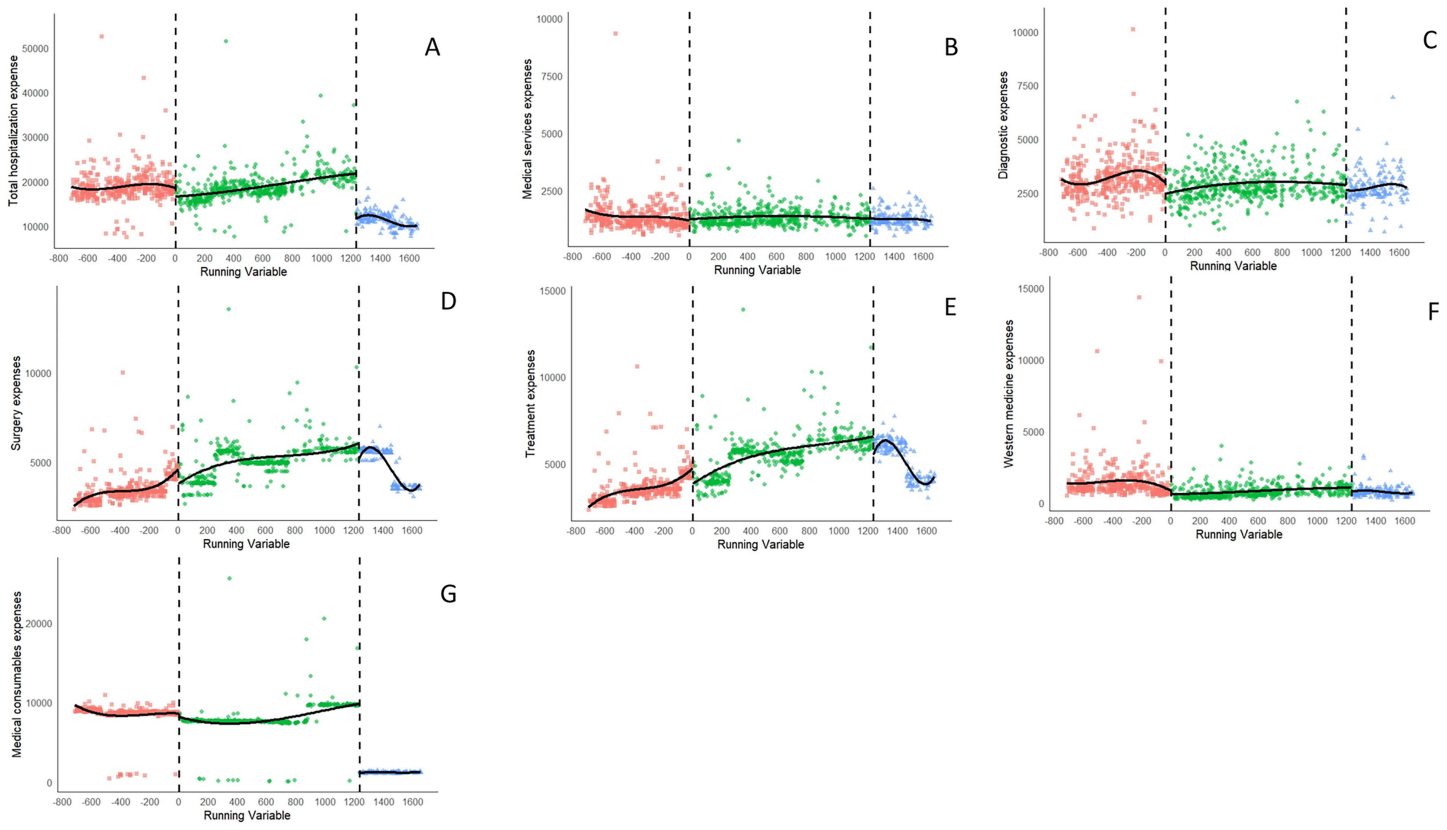
Polynomial fitting diagram of the expenditures after the reform from January 2018 to June 2024. The inpatient expenses include **(A)** total hospitalization expenses, **(B)** medical service expenses, **(C)** diagnostic expenses, **(D)** surgery expenses, **(E)** treatment expenses, **(F)** Western drugs expenses, and **(G)** medical consumable expenses.

**Figure 4:**
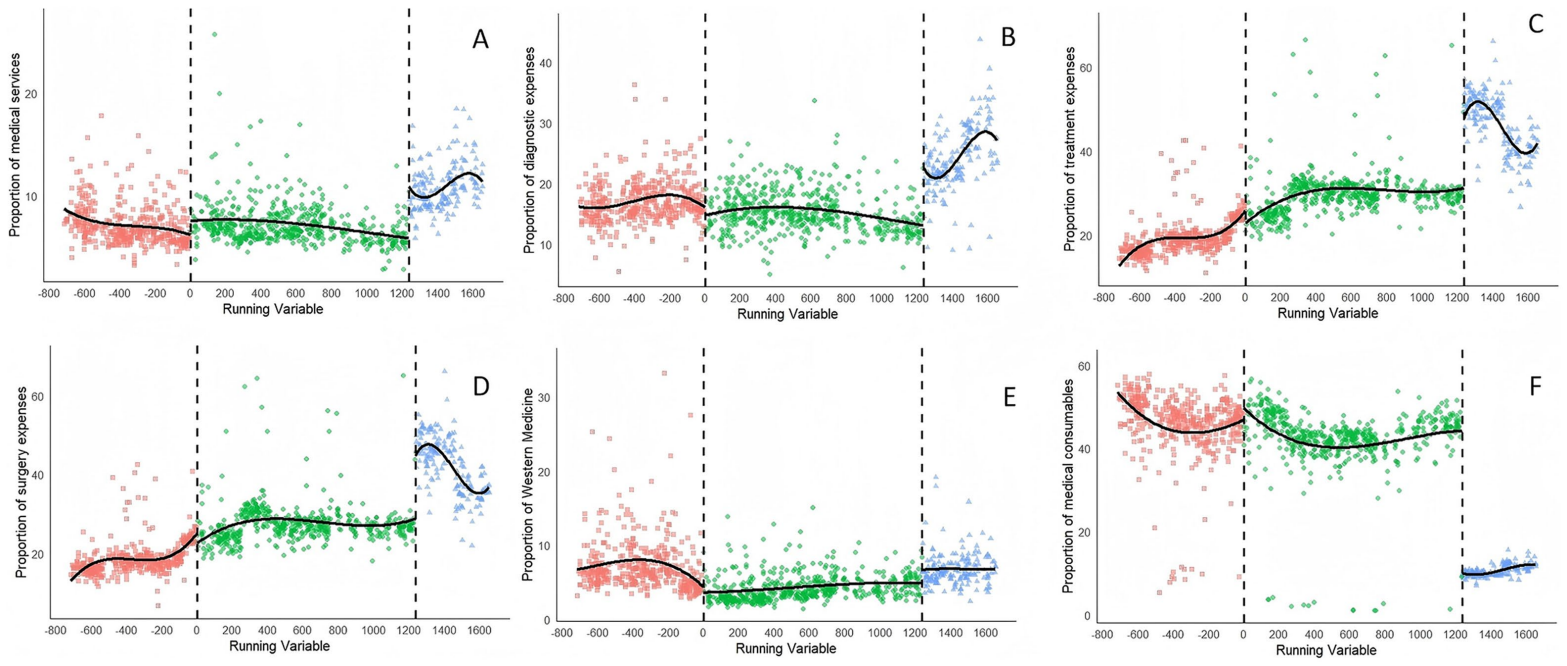
Polynomial fitting diagram of the proportion of expenditures after the reform from January 2018 to June 2024. The proportions include **(A)** medical service expenses, **(B)** diagnostic expenses, **(C)** treatment expenses, **(D)** surgery expenses, **(E)** western drug expenses, and **(F)** medical consumable expenses.

**Figure 5:**
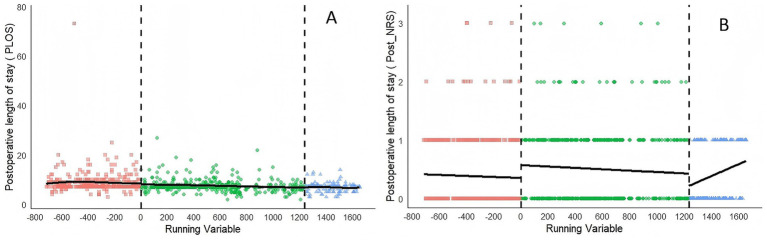
Polynomial fitting diagram of clinical outcomes after the reform from January 2018 to June 2024. **(A)** Postoperative length of stay (post-LOS), **(B)** postoperative NRS (post-NRS).

To ascertain the validity of RDD analysis, a polynomial fitting diagram of the dependent variable was produced (Figures 3–5). Graphical examination confirms continuity at cutoff points, with no discernible discontinuities or anomalies in the data distribution.

Table 3 provides the RDD estimates of the causal impacts of two reform phases on hospitalization expenses, and expenditure proportions, while accounting for covariate effects. The hospitalization costs were log-transformed, and the table’s coefficients show estimated discontinuity at the cutoff, converted into percentage changes for better interpretability of the policy reform’s local effect on expenditures. The number of observations per cutoff point show a balance between pre-reform and post-reform groups. For instance, there are 88 and 61 observations for Reform1, and 48 and 54 for Reform 2 in total hospitalization expenses. This balance supports local comparability and the causal interpretation of RDD estimates.

**Table 3:** The impact of the medical consumable policy on the medical expenses.

Outcomes		Reform phase 1					Reform phase 2				
		Bandwidth	Coefficient	Percentage change (%)	Obs. (L/R)	*P*-value	Bandwidth	Coefficient	Percentage change (%)	Obs. (L/R)	*p*-value
Medical Expenses	Total hospitalization expenses	145.07	−0.033	−3.20	88/61	0.342	107.804	−0.562^#^	−43.00	48/54	<0.001^*^
	Medical service expenses	160.67	0.122^#^	13.00	95/72	0.002^*^	167.890	−0.090	−8.60	65/79	0.030*
	Diagnostic expenses	173.00	−0.100	−9.50	100/84	0.149	116.224	−0.229	−20.50	51/61	0.024^*^
	Treatment expenses	176.02	−0.114	−10.80	102/87	0.038	114.736	−0.072	−6.90	51/59	0.153
	Surgery expenses	187.92	−0.103^#^	−9.80	106/90	0.058	119.878	−0.056^#^	−5.40	52/62	0.235
	Western medicine expenses	157.89	−0.197^#^	−17.90	90/72	0.110	125.078	−0.231	−20.60	53/66	0.079
	Medical consumable expenses	170.07	0.034^#^	3.50	99/84	0.686	227.152	−1.948^#^	−85.40	77/100	<0.001^*^
Proportion of Expenses	Proportion of medical services expenses	146.76	0.945	–	89/61	<0.001	119.721	3.977^#^	–	52/62	<0.001^*^
	Proportion of diagnostic expenses	160.68	−0.707	–	95/72	0.443	85.815	5.389^#^	–	36/44	0.015^*^
	Proportion of treatment expenses	164.14	−1.233^#^	–	95/78	0.377	87.932	18.849^#^	–	39/44	<0.001^*^
	Proportion of surgery expenses	167.81	−0.915^#^	–	98/78	0.497	87.799	18.360^#^	–	39/44	<0.001^*^
	Proportion of Western medicine	192.52	−0.451^#^	–	106/90	0.461	138.693	1.796^#^	–	54/72	0.018^*^
	Proportion of medical consumables	183.04	1.422^#^	–	104/89	0.429	160.253	−32.681^#^	–	63/74	<0.001^*^

Estimates control for sex, age, LOS. Percentage change was defined as [exp (coefficient) - 1] *100. (L/R) denotes the number of observations to the left/right of the cutoff within the data-driven optimal bandwidth. ^*^p < 0.05; ^#^ Indicates variable passing bandwidth sensitivity test (Supplementary Table S3 for details).

The analysis revealed that total hospitalization expenses did not undergo a statistically significant alteration following Reform1 (*β* = −0.033, *p* = 0.342), with this result lacking robustness under narrow bandwidths. However, following Reform2, a pronounced and statistically significant reduction in total hospitalization expenses was observed (*β* = −0.562, *p* = 0.001), which corresponds to a decrease of approximately 43.0%. Medical service expenses initially rose significantly post-Reform 1 (*β* = 0.122, *p* = 0.002), representing a 13.0% increase, then fell after Reform 2 (*β* = −0.09, *p* = 0.030). Changes in surgery and Western medicine expenses did not achieve statistical significance following either reform (*p* > 0.05), suggesting that the two medical consumables pricing policies had limited efficacy in influencing these costs for inpatients. Conversely, medical consumables expenses showed no significant change following Reform1 (*β* = −0.034, *p* = 0.686). However, they exhibited the most substantial decrease after Reform 2 (*β* = −1.948, *p* < 0.001), which corresponds to a cost reduction of approximately 85.4% and indicates the effective cost containment achieved by the CVBP policy for inpatients. In summary, after Reform1, non-statistically significant coefficients observed across various expenditure categories (diagnostic expenses, treatment expenses, surgery expenses, and Western medicine expenses; *p* > 0.05) may imply that the implementation of the ZMCP had a limited impact. Conversely, after Reform2, the consistently statistically significant coefficients across most expenditure categories (total hospitalization expenses and medical consumables expenses, *p* < 0.05) provide robust evidence of the reform’s efficacy in reducing costs. Bandwidths, naturally varying according to the data density specific to each outcome, remained within plausible ranges (107–227 days). The analyses reveal a shift in spending patterns due to the CVBP reform, which contained costs in medical consumables while restructuring inpatient costs.

The coefficients in Reform Phase2 show a major restructuring of medical expenditures after policy implementation. The proportion of medical service expenses exhibited a significant increase following the implementation of Reform2 (*β* = 3.977, *p* < 0.001). In contrast, the results post-Reform1 were not robust, indicating a less discernible impact on service utilization attributable to the CVBP policy. The apparent lack of immediate impact of Reform 1 on most cost components (e.g., treatment, surgery, medicines, and consumables; *p* > 0.05) could be attributed to limited policy penetration or the presence of countervailing institutional factors. On the other hand, the consistent and significant coefficients across all categories after Reform2 (*p* < 0.05) with robust estimates strongly suggest the reform’s effectiveness. Notably, the proportion of medical consumables decreased significantly after Reform2 (*β* = −32.681, *p* < 0.001), reflecting the enforcement of stringent controls on the prices of medical consumables. The proportions of medical service expenses, diagnostic expenses, treatment expenses, surgery expenses, and Western medicine expenses all demonstrated significant up trends (β = 3.977, 5.389, 18.849, 18.360, 1.796; *p* < 0.05,respectively) with robust estimates. The bandwidths varied naturally according to the outcome-specific data density, yet remained within plausible ranges (85–192 days). This change, driven by CVBP reform, shows a drop in consumables and an increase in treatment and surgery expenses.

#### 3.1.4 Results of robustness checks

To validate the reliability of the RDD estimates and address potential identification concerns, we conducted a series of robustness checks.

##### 3.1.4.1 Continuity of the running variable and covariates

McCrary tests were conducted to investigate potential sorting behaviors around the cutoff points (C₁ and C₂). The pre-specified criterion for passing this test was the absence of a statistically significant discontinuity (*p >* 0.05) in the density of the running variable at the cutoff. The results for C₁ (T = −0.957, *p* = 0.338) and C₂ (T = −1.593, *p* = 0.111) did not allow us to reject the null hypothesis of continuity. These findings suggest no evidence of strategic manipulation around the cutoffs, as further supported by the continuity of covariates at the breakpoint according to the density plot (Supplementary Figure S2). This confirms the validity of the RDD by ruling out the possibility that any observed discontinuity in the outcome variable at the breakpoint resulted from changes in covariates or the running variable.

##### 3.1.4.2 Sensitivity to bandwidth selection

Sensitivity to bandwidth selection evaluated the stability of our findings, with robustness defined by the coefficient’s statistical significance and consistent sign across bandwidths. Bandwidth sensitivity analyses were performed using bandwidth settings of 100 and 200, with the results presented in Supplementary Table S3. Variables that met the criteria for robustness are indicated in Table 3. Under Reform1 (ZMCP) initiative, statistical significance was preserved for the coefficients of all seven investigated variables: surgery expenses, Western medicine expenses, medical consumable expenses, proportion of treatment expenses, proportion of surgery expenses, proportion of Western medicine expenses, and proportion of medical consumables expenses. In the context of Reform2 (CVBP), statistical significance was likewise maintained for the coefficients of all nine variables included: total hospitalization expenses, surgery expenses, medical consumables expenses, proportion of medical services expenses, proportion of diagnostic expenses, proportion of treatment expenses, proportion of surgery expenses, proportion of Western medicine expenses, and proportion of medical consumables expenses. However, other variables either demonstrated a reversal in sign or a loss of statistical significance under alternative bandwidth settings, suggesting limited robustness for these outcomes. Variables with changes in sign or lost significance across different bandwidths are deemed less robust. Therefore, they warrant cautious interpretation.

##### 3.1.4.3 Placebo tests on false cutoffs

We conducted placebo tests using false cutoff points—artificial thresholds not corresponding to actual intervention dates—to rule out time trends or unobserved confounders affecting the results. (Supplementary Table S4). These results provided substantial evidence supporting the validity of the primary analysis: 12 out of 13 variables exhibited no statistically significant discontinuities (*p* > 0.05) at the placebo cutoff points, aligning with the null hypothesis that posits an absence of spurious treatment effects. Notably, the variable pertaining to medical services expenses demonstrated a statistically significant discontinuity in the placebo test, necessitating further investigation and warranting caution in the interpretation of these findings.

### 3.2 Qualitative study

The quantitative study revealed significant reductions in both total hospitalizations and costs associated with medical consumables for orthopedic spinal patients. However, the impacts of these cost reductions on hospitals and physicians have not been fully elucidated. To bridge this gap, the present study adopted a qualitative research methodology to further interpret the quantitative results and to explore the implications of the underlying policies.

The semi-structured interviews were conducted (36) and thematic analysis was performed with a stakeholder theory framework being employed. The analysis revealed that the medical consumables pricing policy impacts stakeholders differently depending on their respective roles. Hospitals prioritizing value-based procurement, strive to balance revenue generation with the quality of care whereas patients seek cost-effective treatment outcomes (Table 4). Public hospitals are engaged in enhancing economic efficiency and service quality. Clinicians are tasked with balancing income sustainability with the provision of high-quality care to achieve effective patient satisfaction. Patients, as the primary beneficiaries and key users of the policy, seek access to affordable and high-quality medical services.

**Table 4:** Stakeholders of the medical consumables reform policy.

Stakeholder	Primary objectives	Role in policy ecosystem
Public Hospitals	Enhance economic efficiency and improve service quality while simultaneously advancing institutional development and ensuring patient satisfaction.	Policy implementer • Purchaser
Clinicians	Balance the preservation of income with the provision of high-quality care to achieve patient satisfaction.	Policy Implementer • User
Patients	Access high-quality medical services at affordable costs.	Policy beneficiary • User

#### 3.2.1 Data collection and coding procedure

The study involved seven key informants in qualitative interviews categorized into three distinct stakeholder groups: public hospital administrators (*n* = 2, 28.57%), clinicians (*n* = 2, 28.57%), and patients (*n* = 3, 42.86%). The composition of the sample was 71.43% female and 28.57% male. Participant ages ranged from 35 to 60 years with 42.86% aged between 35 and 40 years, and 57.14% between 41 and 60 years. Detailed demographic characteristics are presented in Supplementary Table S5.

The coding of interview transcripts began with open coding, from which 70 free nodes were derived. Subsequent axial coding of these nodes generated eight tree-like structures, facilitating the emergence of three core categories. The distribution of free nodes across each core category and within each tree-like structure is documented in Supplementary Table S6 and Supplementary Figure S3. The identified thematic categories encompassed hospital economic operation management, the influence on clinical decision-making, and patient decision-making.

#### 3.2.2 Result of qualitative study

##### 3.2.2.1 Hospital financial impact of cost-reduction policies

The qualitative analysis corroborates the cost reductions observed in the quantitative study, strengthens its findings, and provides a deeper insight into the policy’s impact on the operational economics of hospitals. The analysis highlighted 29 instances pertaining to the economic operation management of hospitals influenced by the cost reductions. Three sub-themes were discerned: dual financial- operational pressures (4/29), challenges in managing medical economics (13/29), and counter strategies with recommendations (12/29). Key sources of financial and operational pressures included diminished medical revenues and escalated operational costs linked to consumables management. These were compounded by the synergistic challenges posed by public health crises, such as the COVID-19 pandemic.

The qualitative data suggest that the increase in service charges has not sufficed to offset the revenue deficits experienced by hospitals. This finding is consistent with the lack of a statistically significant increase in surgical and medical service costs as identified in the quantitative analysis. These findings underscore the necessity of implementing systemic improvements to achieve financial and operational sustainability, particularly through a structural income transformation of income toward value-based care.

Interview analyses identified several key strategic responses to the economic challenges in healthcare: cost containment, precision management, value-based service delivery, interdisciplinary collaboration, and dynamic pricing mechanisms.

##### 3.2.2.2 Policy-driven shifts in clinical decision-making for orthopedic spinal care

Qualitative research methodologies offer valuable insights into complex phenomena by uncovering the deeper dimensions often obscured in quantitative analyses. These include stakeholders’ clinical perceptions and the underlying mechanisms of healthcare policies. Our qualitative analysis specifically addressed the shifts in clinical decision-making processes influenced by the consumables policy. Two sub-themes emerged: multidimensional selection decisions regarding medical consumables (10/14) and adjustments to clinical therapeutic regimens (4/14).

Clinicians prioritized clinical efficacy and regulatory compliance as key determinants, while payment constraints (such as insurance coverage limits) became secondary considerations in the selection of medical consumables reform. Notably, patient preferences were considered in only 7% of the references (1/14), highlighting the persistence of provider-dominated decision-making processes.

The policy influenced clinical decision-making by prompting treatment substitutions and reducing surgical motivation due to income disincentives. Although there were increases in service prices, these adjustments did not fully compensate for the reduced costs of medical consumables, leading to financial shortfalls. These economic pressures may drive clinicians to select higher-cost treatments and reduce surgeons’ willingness to operate, owing to diminished performance-based incentives. This observation is consistent with our quantitative findings, which indicated no significant increase in medical service fees, and further exposes the unintended adverse effects of the policy.

##### 3.2.2.3 Concerns regarding clinical outcomes in orthopedic spinal patients

Emerging from the qualitative interviews, a primary concern was that the changes in medical consumables could compromise the efficacy of treatments. However, our quantitative analysis of postoperative outcomes provided a reassuring counterpoint: both the post-LOS and post-NRS scores remained stable throughout the period of reform, suggesting no deterioration in the effectiveness of the consumables used. In addition, three sub-themes were identified: patient awareness of policy channels (13/27), patient involvement in decisions regarding medical consumables (7/27), and policy awareness (7/27). Despite the utilization of multiple dissemination channels, such as hospital notices, physician explanations, and social media, patient awareness of national healthcare policies remains limited. When selecting high-cost medical supplies, patients prioritized quality and effectiveness, followed by affordability.

### 3.3 Integration of quantitative and qualitative findings

The integration of quantitative and qualitative findings demonstrated explanatory complementarity. The mixed-methods design allowed us to construct a coherent narrative that connects statistical outcomes with stakeholder experiences.

First, Quantitative analysis revealed a significant reduction in total hospitalization and consumable expenses. There was no corresponding increase in service or surgery expenses. Qualitative data elucidated the institutional implications of this financial trend. Hospital administrators and clinicians reported substantial operational strain resulting from declining consumable revenues due to inadequate reimbursement. This finding corroborates and contextualizes the quantitative results. Moreover, this financial pressure directly drives the institution’s efforts to contain costs. Furthermore, the qualitative analysis revealed behavioral adaptations not captured by quantitative methods. As a response to pressures to contain overall patient expenditures, physicians increasingly adopted simpler surgical procedures. This indicates that cost reduction was achieved through substitution and efficiency gains without compromising necessary care, thereby preserving patient outcomes. Finally, the mixed-methods design resolved an apparent paradox: while quantitative analysis of clinical outcomes (e.g., post-NRS, post-LOS) confirmed the stability of treatment efficacy following the reform, qualitative interviews revealed patients’ concerns regarding potential compromises in care quality. The quantitative findings alleviated these concerns, providing a more comprehensive understanding of the impact of the reforms. Meanwhile, the qualitative data identify a critical area for improvement in patient-centered communication.

## 4 Discussion

In an effort to control the growth of healthcare expenditures, the Chinese government has eliminated markups on medical consumables and introduced CVBP for high-value consumables in public hospitals. These initiatives were part of the reforms carried out in 2019, focusing on ZMCP, and in 2023, concentrating on CVBP for orthopedic spinal procedures in the third batch. This study utilized RDD and semi-structured interviews to assess the impact of the ZMCP and CVBP policies on hospitalization costs and clinical outcomes for orthopedic spinal patients. It also aimed to identify challenges associated with policy implementation.

The findings indicate that the CVBP reforms were associated with significant reductions in total hospitalization (37) and consumable costs. Conversely, the ZMCP appeared to have a limited effect on costs. Clinical outcome metrics, such as post-LOS and post-NRS, remained relatively stable and did not show clear variations attributable to the policy changes. Subsequent analysis indicated that the CVBP policy may have influenced the restructuring of cost proportions more noticeably than the ZMCP policy. The qualitative phase of the study highlighted several unintended consequences related to the medical consumables policy, including its potential influence on clinical decision-making, pressures on hospital economic sustainability, and concerns among orthopedic spinal patients (38). These interviews helped to contextualize and expand the findings from the quantitative analyses.

These results demonstrate that the mixed-methods design not only enriches and reconciles empirical evidence but also provides a comprehensive understanding. The quantitative data confirm the reforms’ effectiveness in containing costs without compromising clinical outcomes, while the qualitative findings reveal the underlying institutional pressures, adaptive clinical behaviors, and patient perceptions that contextualize these results. The analysis shows cost reduction mainly from efficiency gains and procedural changes, ensuring clinical safety, rather than through reductions in necessary care. This study clarifies healthcare policy complexities in China, highlights implementation challenges, and offers initial evidence of outcomes and underlying mechanisms. While RDD strengthens causal inference, the results may be biased due to unobserved time-varying confounders. When generalizing the findings, limitations related to the single-center design, temporal scope, and potential unobserved confounders should be considered.

### 4.1 Financial impact on hospitals of orthopedic spinal devices policies

The study suggests the program’s effectiveness in reducing both hospitalization costs and expenses for consumables. Following the implementation of the CVBP, total hospitalization costs for orthopedic spinal patients decreased significantly. The log-transformed reduction was 0.562, corresponding to a 43% cost reduction. Similarly, medical consumable expenses decreased after CVBP. The log-transformed dropped by 1.948, representing an 85% cost reduction. In contrast, medical services expenses increased significantly after the introduction of ZMCP. Overall, the policy effectively contained material costs while promoting the appropriate valuation of medical services (39). The RDD indicated that the CVBP policy had a greater impact on the restructuring of cost proportions of healthcare costs compared to the ZMCP policy. The proportions of consumables decreased by 32.68% following the implementation of CVBP, but did not change significantly after the implementation of ZMCP. This reflects the structural changes observed in Taiwan’s global budget system (40), indicating a common feature of pricing reforms. Notably, the proportions of treatment, surgery, and Western medicine expenses did not decrease significantly after ZMCP. However, after CVBP, these proportions increased significantly by 18.85, 18.36, and 1.80%, respectively. While these costs did not increase significantly in absolute terms, their shares relative to total expenses rose significantly due to the overall reduction in hospitalization costs under CVBP.

The qualitative data supported the quantitative evidence indicating a decrease in the costs of medical consumables. Additionally, these data revealed a significant financial burden imposed on providers of orthopedic spinal care by these policies. The hospitals have faced dual pressures: revenue losses from the elimination of markups on consumables and rising operational costs. The prevalence of medical revenue shortfall underscores systemic vulnerabilities, as service price adjustments have failed to compensate for revenue losses (2). Insufficient government subsidies have further exacerbated financial disparities, among providers, highlighting the need for targeted income diversification strategies based on value. This situation also aligns with WHO’s warnings regarding the risks associated with phased reimbursement reforms, when implemented without transitional support (41). Moreover, the pandemic has further strained resources, reflecting global reports of healthcare financial fragility.

### 4.2 Impact on clinical outcomes of orthopedic spinal patients

The quantitative analysis of postoperative outcomes provided evidence that both post-LOS and post-NRS remained stable throughout the reform period, indicating no deterioration in the effectiveness of the consumables used. The consistent clinical outcomes indicate that the therapeutic efficacy of devices regulated by procurement policies was maintained. Neither pain management nor recovery duration was negatively affected, despite significant cost reductions. Regarding the evaluation of treatment effectiveness, qualitative interviews revealed that patients raised concerns that the national procurement policy might undermine the treatment efficacy, as they have limited involvement in decisions about medical consumables. Concerning the findings, physician counseling has been the primary source of policy information; however low awareness and financial limitations have largely influenced patient choices. Institutional media have underperformed in raising awareness, highlighting the necessity for patient-centered aids (42).

### 4.3 Exploring the mechanisms behind policy-induced changes in clinical practice

The qualitative study revealed the potential mechanisms driving these observed changes. The policies influenced clinician behavior through trade-offs in the selection of consumables and therapeutic regimens, an effect not originally anticipated by policymakers. This finding implies that hospitals, within the constraints of the reimbursement framework, sought to offset decreases in consumables revenue by either adopting non-surgical alternatives or opting for consumables with higher profit margins. This behavior corroborates previous studies indicating that reductions in fee-for-service payments may encourage defensive medicine (43). Significantly, the policy fostered treatment substitutions, such as transitions to other surgical options—a trend observed in other fee-for-service systems (44). This study demonstrated the income-driven elasticity of clinical decisions, whereby diminished revenue from consumables discourages certain procedures. These unintended consequences underscore the necessity of reforming physician compensation structures to better align financial incentives with clinical outcomes. Such alignment is consistent with the development of metrics that effectively align clinical incentives with institutional goals (45), as well as the aligning physician compensation with strategic goals (46).

Overall, this study offers insights into healthcare system reforms. First, a dynamic price adjustment mechanism for healthcare services should be established, using feedback from facilities and corresponding cost calculations (10). However, under the prevailing fee-for-service models, surgeons face conflicting priorities. While the hospital aims to reduce expenditures on consumables as stipulated by policies, surgeons are motivated to increase fees for technical procedures (47).

### 4.4 International comparative perspectives

Compared with international counterparts, certain aspects of the reform may remain less effective. Moreover, our finding that the CVBP policy imposed significant financial strain on hospitals is consistent with early implementations of similar centralized procurement models in the United States (48). The U. S. Group Purchasing Organization (GPO) model provides subsidies. In contrast, China lacks subsidy programs like in the U. S. GPO model, so, it increases financial pressure on interviewees. Chinese CVBP shares a philosophy with the United Kingdom’s National Health Service (NHS) (49) in centralized negotiation, however, its implementation varies. The Chinese zero-markup reform created a revenue gap that requires financial compensation. In contrast, the NHS uses competitive pricing in contracts to avoid markups. Additionally, Chinese hospitals face financial stress, unlike in Japan, where biennial price cuts are absorbed by the insurance system, thereby minimizing the financial impact on providers (50). This comparison underscores that cost containment must be accompanied by systemic supports, such as institutional and policy adjustments, to ensure sustainability. Effective price negotiation alone is inadequate; it must be combined with these system-level measures. In conclusion, successful centralized procurement requires pairing price negotiation with comprehensive institutional support to maintain financial stability.

### 4.5 Research highlights and limitations

This study contributes to health policy evaluation in two key ways. First, it employs a rigorous analysis with robustness checks to ascertain the effects of medical consumables reforms. Second, this study employs a mixed-methods approach, combining quantitative analysis with stakeholder interviews to reveal overlooked aspects of reform implementation. These include unintended policy-driven shifts in clinical decision-making for orthopedic spinal care and financial pressures on hospitals. These methodological and substantive innovations provide researchers and policymakers with robust tools and actionable insights for evaluating and implementing medical pricing reforms in across healthcare systems.

Several limitations of this study warrant attention. Firstly, the single-center, mixed-methods design inherently limits the external validity and applicability of the findings to broader populations and different settings. When interpreting these findings, it is essential to consider regional variations in economic conditions and healthcare needs. Therefore, we urge caution against excessive generalization. Secondly, the robustness checks for certain outcome variables of the RDD, such as medical service expenses, failed to meet conventional statistical significance thresholds. Consequently, it was not feasible to establish definitive conclusions about the policy’s causal effects on these metrics. This limitation may stem from either inadequate statistical power or the presence of unobserved confounding factors that independently influence these outcomes, irrespective of the policy intervention. Thirdly, unmeasured confounders, such as concurrent Diagnosis Related Groups (DRG) pilots or changes in management strategies, could introduce biases despite rigorous RDD implementation. Furthermore, the reliability of the qualitative interviews may be limited by recall bias, where participants recounted past events, and reporting bias, where they provided answers they perceived as favorable. Although anonymity was assured to mitigate these biases, their potential influence cannot be fully eliminated. These limitations underscore the necessity for future studies with multi-center mixed-methods designs and extended follow-up periods. Future research involving larger samples or additional quasi-experimental designs could provide greater clarity on these relationships.

## 5 Conclusion

The reforms in medical consumables in China appear to have effectively controlled costs. However, current physician compensation models are driven by volume, so complementary payment reforms are necessary. Furthermore, these reforms highlight inherent systemic tensions between price regulation and the sustainable provision of services. These findings underscore the urgent need for integrated reforms integrating effective price negotiation and a comprehensive systemic support framework. Specifically, key measures should include implementing transitional subsidies, reforming physician compensation to reflect the actual value of medical services provided, and ensuring the overall financial sustainability of healthcare institutions.

## Data Availability

The raw data supporting the conclusions of this article will be made available by the authors, without undue reservation.
