# Supplementary material for: Impact of zero-markup consumable policy and national procurement of orthopedic spinal devices in China: a mixed-methods study
Source: Front Public Health. 2026 Jan 21;13:1715856. doi: 10.3389/fpubh.2025.1715856 (PMC12868270; doi:10.3389/fpubh.2025.1715856)
Supplement: Supplementary file 1 [file Data_Sheet_1.docx]

**Supplementary materials**

Table

Table S1. Semi-Structured interview guide:Impact of for medical consumables policy

Table S2. McCrary Density Test for Manipulation of Running Variable

Table S3. Robustness Checks with Alternative Bandwidths

Table S4. Placebo Tests for Regression Discontinuity Design

Table S5. Characteristics of interviewed key informants

Table S6. Thematic categories and frequency of reference points from interviews

Figure

Figure S1. Flow Chart of Participants

Figure S2. Density map of LOS and age

Figure S3. The node-level diagram of the interview record

January 2018 to June 2024

lumbar disc herniation patients

N=2811

Included N=1125

Excluded

Not percutaneous endoscopic lumbar discectomy

N=1686

Excluded

Incomplete information

N=26

N=?

Participant N=1099

Figure S1. Flow Chart of Participants


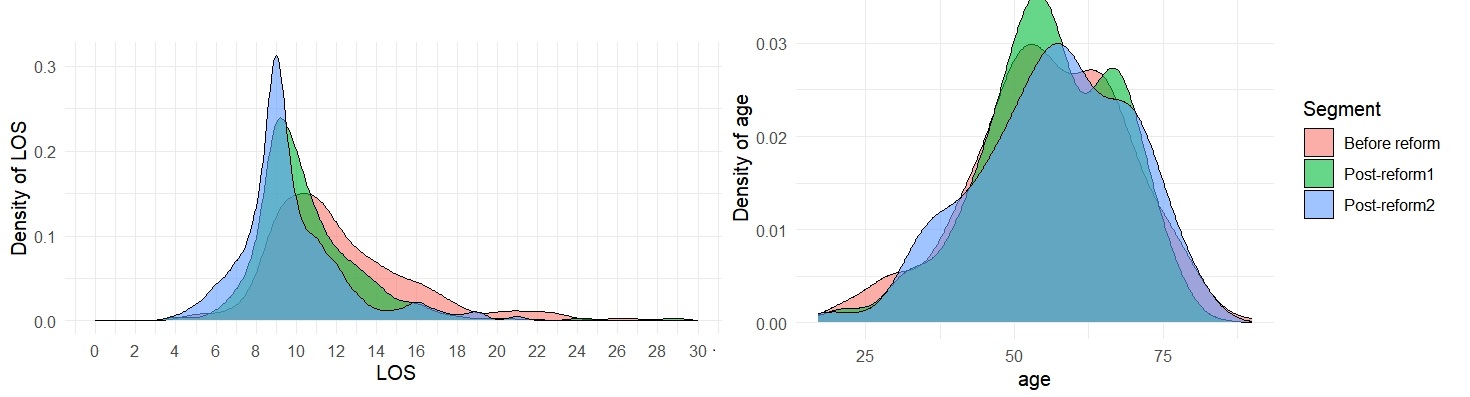
Figure S2. Density map of LOS and age


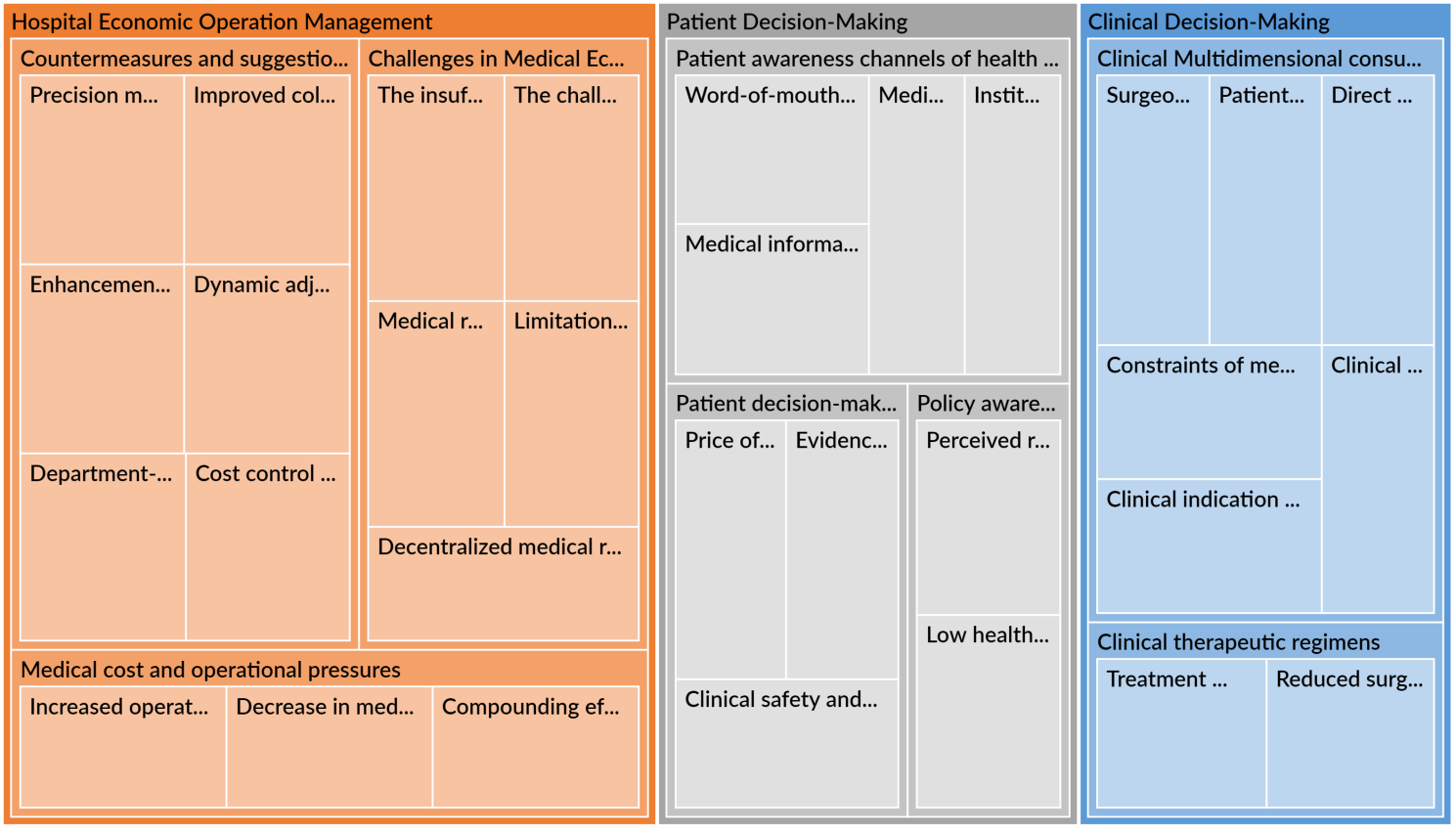


Figure S3. The node-level diagram of the interview record

### Table S1. Semi-Structured interview guide: Impact of for medical consumables policy

| Interview guide for medical consumable policy general information  Participant ID: |
| --- |
| Background Information  Gender: Age: Identity: |
| **Introduction**  Hello, I’m a researcher from Shandong Second Medical University, and I am conducting a study on medical consumable policy in China. I wish to hear from you about consumable policy in China; may I bother you for an interview?  **Informed consen**t  All the information you provide will be used for research purpose only, and your name and other personal information will not appear in the results. Do you mind participating in this interview? Do you have any questions? |
| I. Clinicians: Policy Impact on Departments and Individuals  1.1 How has the adjustment of medical consumables policies affected your department’s operations after implementation ?  1.2 From your perspective, what impact has the policy had on physicians’ work motivation and income ?  1.3 What factors do you prioritize when selecting consumables ? |
| II. Administrators: Impact on Institutional Operations  2.1. Financial Managers  2.1.1 How has policy implementation affected hospital operations? What mitigation measures have been adopted ?  2.1.2 Have these measures fully offset the financial losses caused by the elimination of consumables markup ?  2.1.3 What further improvements are needed in management practices ?  2.2 Consumables Managers  2.2.1 How has the policy impacted consumables management ?  2.2.2 What strategies have been implemented for cost control ?  2.2.3 What areas require improvement in future consumables management ? |
| III. Patients: Policy Awareness and Experience  3.1 Have you noticed changes in medical consumables costs before and after the policy? Through which channels did you learn about the policy ?  3.2 Did your physician adequately explain the differences between consumables (e.g., price, brand, performance) before selection ? If required to self-pay for higher-cost options (e.g., imported materials), how would you balance price and quality ? |

Table S2. McCrary Density Test for Manipulation of Running Variable

| Cutoff Point | Side of Cutoff | Observations | Test Statistic (T) | P-value |
| --- | --- | --- | --- | --- |
| C1 | left | 399 | -0.957 | 0.338 |
|  | right | 700 |  |  |
| C2 | left | 933 | -1.593 | 0.111 |

Table S3. Robustness Checks with Alternative Bandwidths

| Outcome | Bandwidth | post-reform 1 | | post-reform 2 | |
| --- | --- | --- | --- | --- | --- |
|  |  | Coefficient | *P*-value | Coefficient | *P*-value |
| Total hospitalization expense | Small(H=100) | 0.006 | 0.879 | -0.559 | <0.001 |
|  | Large(H=200) | -0.044 | 0.147 | -0.547 | <0.001 |
| Medical services expenses | Small(H=100) | 0.102 | 0.026 | -0.062 | 0.207 |
|  | Large(H=200) | 0.134 | <0.001 | -0.084 | 0.028 |
| Diagnostic xpenses | Small(H=100) | 0.044 | 0.648 | -0.234 | 0.033 |
|  | Large(H=200) | -0.125 | 0.053 | -0.139 | 0.084 |
| Treatment xpenses | Small(H=100) | -0.036 | 0.507 | -0.076 | 0.149 |
|  | Large(H=200) | -0.116 | 0.032 | -0.055 | 0.143 |
| Surgery expenses | Small(H=100) | -0.025 | 0.629 | -0.058 | 0.249 |
|  | Large(H=200) | -0.102 | 0.060 | -0.043 | 0.221 |
| Western medicine expenses | Small(H=100) | -0.054 | 0.755 | -0.200 | 0.167 |
|  | Large(H=200) | -0.174 | 0.110 | -0.271 | 0.008 |
| Medical consumables expenses | Small(H=100) | 0.007 | 0.939 | -1.944 | <0.001 |
|  | Large(H=200) | 0.028 | 0.718 | -1.945 | <0.001 |
| Proportion of medical services expenses | Small(H=100) | 0.639 | 0.082 | 4.027 | <0.001 |
|  | Large(H=200) | 1.091 | 0.001 | 3.839 | <0.001 |
| Proportion of diagnostic expenses | Small(H=100) | 0.655 | 0.582 | 5.532 | 0.003 |
|  | Large(H=200) | -1.230 | 0.137 | 6.996 | <0.001 |
| Proportion of treatment expenses | Small(H=100) | -0.843 | 0.599 | 19.444 | <0.001 |
|  | Large(H=200) | -1.293 | 0.328 | 19.303 | <0.001 |
| Proportion of surgery expenses | Small(H=100) | -0.554 | 0.715 | 18.685 | <0.001 |
|  | Large(H=200) | -0.886 | 0.491 | 18.219 | <0.001 |
| Proportion of western medicine | Small(H=100) | -0.047 | 0.961 | 1.915 | 0.042 |
|  | Large(H=200) | -0.391 | 0.517 | 1.709 | 0.004 |
| Proportion of medical consumables | Small(H=100) | 0.982 | 0.639 | -32.173 | <0.001 |
|  | Large(H=200) | 1.339 | 0.439 | -33.126 | <0.001 |
| Post_NRS | Small(H=100) | 0.145 | 0.540 | -0.278 | 0.195 |
|  | Large(H=200) | 0.087 | 0.613 | -0.057 | 0.732 |
| PLOS | Small(H=100) | -0.465 | 0.263 | 0.230 | 0.642 |
|  | Large(H=200) | -0.488 | 0.161 | 0.076 | 0.843 |

Note: Estimates control for sex, age, LOS.

Table S4. Placebo Tests for Regression Discontinuity Design

| Outcome Variable | Coefficient | Conventional *P*-value | Robust *P*-value |
| --- | --- | --- | --- |
| Total hospitalization expense | 0.01 | 0.812 | 0.612 |
| Medical services expenses | 0.075 | 0.011 | 0.008* |
| Diagnostic expenses | 0.005 | 0.915 | 0.773 |
| Treatment expenses | -0.052 | 0.018 | 0.076 |
| Surgery expenses | -0.026 | 0.262 | 0.305 |
| Western medicine expenses | 0.111 | 0.091 | 0.070 |
| Medical consumables expenses | 0.063 | 0.843 | 0.758 |
| Proportion of medical services expenses | 0.074 | 0.124 | 0.230 |
| Proportion of diagnostic expenses | 0.032 | 0.599 | 0.715 |
| Proportion of treatment expenses | -0.046 | 0.250 | 0.336 |
| Proportion of surgery expenses | -0.018 | 0.722 | 0.919 |
| Proportion of western medicine | 0.165 | 0.060 | 0.075 |
| *Proportion of medical consumables | 0.057 | 0.834 | 0.775 |

**P*<0.05

Table S5. Characteristics of interviewed key informants

| Characteristics | Description | N | % |
| --- | --- | --- | --- |
| Identity | Hospital administrators | 2 | 28.57 |
|  | Clinicians | 2 | 28.57 |
|  | Patients | 3 | 42.86 |
| Gender | Male | 2 | 28.57 |
|  | Female | 5 | 71.43 |
| Age | 35-40 years | 3 | 42.86 |
|  | 41-60 years | 4 | 57.14 |

Table S6. Thematic categories and frequency of reference points from interviews

| Core coding nodes | Tree-like nodes | Free nodes | Quotes | Reference Points |
| --- | --- | --- | --- | --- |
| Hospital Economic Operation Management (29) | Challenges in Medical Economic Operation(13) | Limitations in medical service price adjustments | Adjustments to medical service prices are constrained by policy, limiting the potential for revenue recovery. | 2 |
|  |  | Medical revenue gap effects | Although corresponding measures have been implemented and have partially alleviated financial pressures, they have not fully offset the revenue losses. | 5 |
|  |  | The challenge of income structure transformation | The implementation of the medical consumables policy has transformed hospitals from profit centers into cost centers. | 4 |
|  |  | The insufficiency of government financial subsidies | Insufficient government subsidies have exacerbated the financial burden on hospitals. | 2 |
|  | Countermeasures and suggestions(12) | Cost control enhancement strategies | Hospitals are optimizing procurement and inventory management to reduce unnecessary expenditures. | 5 |
|  |  | Department-specific performance targets | The hospital has revised the utilization targets for medications and consumables across all clinical departments. | 1 |
|  |  | Dynamic adjustment of medical service pricing | The policy should grant greater flexibility in adjusting medical service prices to alleviate the financial strain on hospitals. | 1 |
|  |  | Enhancement of technical service value | Enhancing the value of medical services is crucial for compensating for the hospital's revenue shortfall. | 2 |
|  |  | Improved collaboration between management and clinical departments | Closer collaboration between clinical departments and hospital administration is essential to ensure that consumable selection balances clinical needs with cost-control objectives. | 1 |
|  |  | Precision management of medical consumables | All departments are mandated to ensure the rational use and refined management of pharmaceuticals and consumables. | 2 |
|  | Medical cost and operational pressures(4) | Compounding effects of public health emergencies | The financial strain on hospitals has been exacerbated by limited government subsidies, a situation further compounded by the COVID-19 pandemic. | 1 |
|  |  | Decrease in medical revenue | The abolition of the medical consumables markup has inevitably led to a significant decline in hospital revenue. | 2 |
|  |  | Increased operational costs for consumables management | The stringent controls on consumable utilization have increased administrative complexity and raised operational costs. | 1 |
| Clinical Decision-Making(14) | Clinical Multidimensional consumables selection decisions(10) | Clinical efficacy orientation | The primary criteria for selecting medical consumables are their quality and clinical efficacy | 2 |
|  |  | Clinical indication compliance | The choice of consumables is tailored to the specific disease and its severity. | 2 |
|  |  | Constraints of medical insurance payment policies | Reimbursement policies from the health insurance scheme are another critical factor in our decision-making process. | 2 |
|  |  | Direct costs of medical consumables | The direct cost of the consumables is, of course, a necessary consideration. | 2 |
|  |  | Patient preference integration mechanisms | Patient preference also plays a significant role in the final selection of consumables. | 1 |
|  |  | Surgeons' procedural preferences | The selection of high-value medical consumables, including their category and brand, heavily relies on the preference and clinical judgment of the operating surgeon. | 1 |
|  | Clinical therapeutic regimens(4) | Reduced surgical motivation due to inadequate incentives | The decline in surgical procedure fees has negatively impacted departmental revenue, which in turn has dampened physicians' enthusiasm for performing certain surgeries. | 2 |
|  |  | Treatment modality substitution elasticity | There is a growing tendency to opt for more conservative treatment approaches, partly attributable to the reduced financial incentive associated with surgical interventions. | 2 |
| Patient Decision-Making(27) | Patient awareness channels of health policies(13) | Institutional media releases | Some policy-related information is disseminated through the hospital's official social media account | 3 |
|  |  | Medical information disclosure | Key policy points are also promoted on the hospital's internal announcement screens. | 3 |
|  |  | Medical counseling | Attending physicians sometimes explain the current pricing policies when prescribing consumables. | 6 |
|  |  | Word-of-mouth through social networks | I've heard from my neighbors that their surgical costs were significantly lower than before. | 1 |
|  | Policy awareness and patient experiences(7) | Low health policy literacy rates | I am aware of some policies related to consumable costs, but I am not entirely clear about the specifics. | 4 |
|  |  | Perceived reduction in financial burden | I know that after the policy was implemented, the cost of some high-value consumables dropped noticeably. | 3 |
|  | Patient decision-making on medical consumables(7) | Clinical safety and efficacy considerations | When considering out-of-pocket consumables, safety and treatment outcomes are the foremost concerns. | 3 |
|  |  | Price of the consumables | For particularly expensive out-of-pocket consumables, the patient's financial capacity becomes a major determining factor. | 3 |
|  |  | Evidence-based clinical recommendations | We ultimately defer to the professional recommendation of our physicians regarding the choice of consumables. | 1 |
